# Infant Feeding Practices Among Indigenous Women in Northwest Territories, Canada: Results From the Maternal and Infant Health Project

**DOI:** 10.1002/fsn3.70376

**Published:** 2025-06-18

**Authors:** Rachel Harris, Fariba Kolahdooz, Radha Sharma, Moutasem Zakkar, Adrian Wagg, André Corriveau, Marie Tarrant, Stephanie Irlbacher‐Fox, Tyler Verhaeghe, Sangita Sharma

**Affiliations:** ^1^ Indigenous and Global Health Research Group, Department of Medicine, Faculty of Medicine & Dentistry College of Health Sciences, University of Alberta Edmonton Alberta Canada; ^2^ Division of Geriatric Medicine, Department of Medicine, Faculty of Medicine & Dentistry College of Health Sciences, University of Alberta Edmonton Alberta Canada; ^3^ Independent Public Health Consultant for the Northwest Territories and Nunavut Yellowknife Northwest Territories Canada; ^4^ School of Nursing, Faculty of Health and Social Development University of British Columbia Kelowna British Columbia Canada; ^5^ Hotıì Ts'eeda Northwest Territories SPOR SUPPORT Unit Yellowknife Northwest Territories Canada; ^6^ Mike Petryk School of Dentistry, Faculty of Medicine & Dentistry College of Health Sciences, University of Alberta Edmonton Alberta Canada

## Abstract

This project occurred in three Northwest Territories (NWT) communities and aimed to assess infant feeding practices and experiences among Indigenous mothers. Utilizing a cross‐sectional study design, self‐identifying Indigenous women of childbearing age (15–49 years) who had delivered in the past three years were invited to participate. Quantitative and qualitative data were collected via a semi‐structured questionnaire regarding infant feeding intentions, feeding experiences, and contact with the baby after childbirth. Qualitative data were analyzed using reflexive thematic analysis. Of the 145 participants (mean age 29.78 years; SD ±6.08), 12% were pregnant. Most participants (73%) reported having had the intention to exclusively breastfeed before birth; 87% initiated breastfeeding, with 82% receiving breastfeeding initiation support at the hospital or health centre; and 48% reported no challenges breastfeeding. 76% had skin‐to‐skin contact when holding the baby for the first time. Rates of skin‐to‐skin contact and rooming‐in were significantly higher (*p* < 0.0001) among participants who had vaginal births than among participants who had emergency and scheduled Caesarean ‐sections. Regarding feeding, 44% of participants exclusively breastfed for up to three months, and 50% introduced solid foods between six and twelve months. Traditional foods and medicine were introduced to 66% of infants before twelve months. Three themes emerged: barriers to breastfeeding, facilitators of breastfeeding, and formula feeding. Many women participating in this project successfully initiated breastfeeding and reported a positive experience with no challenges. To further improve infant nutrition in NWT, a community‐led approach leveraging local expertise, traditional knowledge, and the wisdom of community Elders is encouraged.

## Graphical abstract

Eighty‐seven percent of women initiated breastfeeding, 82% received breastfeeding support at the hospital or health center, and 76% had skin‐to‐skin contact after birth. Forty‐four percent exclusively breastfed for up to three months, while 50% introduced solid foods between six and twelve months. To improve infant nutrition in NWT, a community‐led approach leveraging local expertise, traditional knowledge, and Elder wisdom is encouraged.

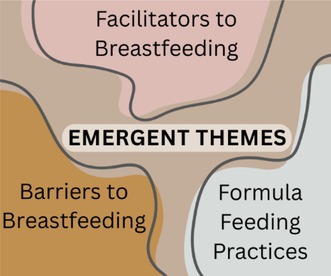

## 1 Introduction

The United Nations (UN) Decade of Action on Nutrition (2016–2025) and the United Nations Sustainable Development Goals highlight the importance of early life nutrition (The UN 2018). Early nutrition for infants (0–12 months) plays an essential role in brain growth (Black 2018), metabolic and biological development (Agosti et al. 2017), and resistance to infection. Receiving suboptimal nutrition in the first 2 years of life can impact an individual's growth, development, and health status throughout life (C. G. Victora et al. 2021).

Health Canada (Health Canada 2012) and the Centers for Disease Control and Prevention (CDC) recommend exclusively breastfeeding infants for the first six months (Eidelman et al. 2012) and continuing breastfeeding up to the age of two years or longer, with appropriate complementary feeding in addition to human milk being introduced at about 6 months of age (Bhutta et al. 2013). In addition to providing nutrition, breastfeeding is an important aspect of population health. For instance, the bioactive and nutrient composition of human milk is beneficial to an infant's immune system and neurocognitive development, and protects against chronic disease development (Yamada and Chong 2017). The role of human milk in an infant's microbiome (Faintuch and Faintuch 2019) and epigenome (Hartwig et al. 2017) development is an additional possible benefit. Breastfeeding can also have dental benefits by preventing the onset of certain types of dental malocclusions (Cenzato et al. 2023). As well, breastfeeding reduces a mother's risk of heart disease, type 2 diabetes, and certain cancers (ovarian and breast cancer) (Moffitt and Dickinson 2016) and improves birth spacing (Cesar G Victora et al. 2016). However, breastfeeding may not always be feasible or solely sufficient for some mothers and infants, and in such cases, infant formula can be introduced as a viable alternative (Martin et al. 2016).

Within Northwest Territories (NWT), one of three Northern territories in Canada with a primarily Indigenous population, the demand for both maternal healthcare and infant nutrition is increasing; in 2021, the fertility rate in NWT for women of child‐bearing age (15–49 years) was 1.6 births per woman, higher than the national rate of 1.43 births per woman in Canada (Statistics Canada 2024). For Indigenous community members, breastfeeding is a sacred and life‐sustaining act that connects mothers to past generations, the home community, and the surrounding environment (Smylie 2014). However, breastfeeding rates among Indigenous communities began to decline in the 1960s (Langner and Steckle 1991). This decline has continued, and remains at suboptimal levels across Canada (McIsaac et al. 2015; McQueen et al. 2015), with breastfeeding initiation rates for Indigenous mothers (77.8%) remaining lower than for non‐Indigenous mothers (87.3%) (Health Canada 2012). Within Indigenous communities, historical colonial policies and the resulting intergenerational trauma have also had a detrimental impact on the knowledge transfer (Dodgson and Struthers 2003) of infant feeding practices, that which were traditionally passed down by experienced mothers, family matriarchs, and community Elders (Smylie 2014).

Many factors can influence feeding intentions and practices. For breastfeeding, barriers include stigma, limited breastfeeding informational and support services, limited maternity leave policies, delayed contact with newborn babies, and non‐vaginal deliveries (Nickel et al. 2014), with mothers who give birth via Caesarean section (C‐section) or assisted vaginal delivery being less likely to exclusively breastfeed (Ogbo et al. 2016). Specific factors influencing the breastfeeding practices of Indigenous mothers include income support, education, and age of the mother (Monteith et al. 2024). The presence of natal (at birth) or neonatal (within the first month) teeth, with the highest incidence occurring in North America, may also be a barrier to breastfeeding due to difficulty with suckling or discomfort experienced by the mother (Vitali et al. 2023).

Breastfeeding is known to have a protective effect against several chronic conditions in childhood and adulthood (Kelishadi and Farajian 2014). Furthermore, chronic health conditions among mothers also affect the initiation and cessation of breastfeeding. A cross‐sectional survey (2015/2016) estimated that the odds of early cessation of exclusive breastfeeding were twice (OR = 2.48, 95% CI 1.49–4.12) among women with chronic diseases compared to women without chronic diseases (Scime et al. 2022). Given the higher rates of non‐communicable chronic conditions among Indigenous women and the disparity in healthcare access, it is important to understand the infant feeding practices among Indigenous mothers in Canada (Cheran et al. 2023; Srugo et al. 2023). Minimal information is available regarding the infant feeding experiences and practices of Indigenous mothers in NWT. This paper addresses that gap in knowledge through quantitative and qualitative inquiry exploring the infant feeding intentions of mothers, the barriers and facilitators to breastfeeding, and complementary feeding practices.

## 2 Materials and Methods

### 2.1 Project Design and Setting

Utilizing a convergent, mixed methods cross‐sectional study design, this paper is part of the Maternal & Infant Health Project, a project aimed at improving maternal and infant health in NWT, Canada (Kolahdooz et al. 2025). This project was conducted in three communities (community A, community B, and community C) with varying levels of access to family programs (Health and Social Services Authority 2021) selected through extensive community engagement, established relationships, and approved participation. Further details have been published elsewhere (Kolahdooz et al. 2025). Most (48%–65%) of the individuals in the communities are Indigenous (NWT Bureau of Statistics 2012, 2013, 2016). This project utilized community‐based participatory research (CBPR) methods (Vaughn and Jacquez 2020) and was planned in partnership with a Community Advisory Board (CAB) comprised of Elders, community members, and local governance organizations.

### 2.2 Recruitment

Self‐identifying Indigenous women of childbearing age (17–49 years) who had given birth in the last three years, and who resided in one of the communities, were invited to participate. This project utilized convenience sampling methods. Local research assistants recruited participants utilizing community‐specific strategies, including via an Indigenous community coordinator, via telephone and community‐based presentations, and by partnering with local community agencies. Passive advertising was also utilized.

### 2.3 Data Collection

Quantitative and qualitative data were collected between October and November 2019, via a semi‐structured questionnaire that included both closed‐ and open‐ended questions, allowing participants to provide more detailed information. The questionnaire was administered by local research assistants at a venue of the participant's choice (local project office, participants' homes, or another private location). The questionnaire design was guided by the CAB to ensure that all questions were culturally relevant and appropriate. Questions regarding sociodemographic and health information, as well as aspects of maternal healthcare, were included. Open‐ended questions were also asked regarding the following subjects: infant feeding intentions during pregnancy; breastfeeding initiation and contact with the baby after birth (skin‐to‐skin, rooming in); breastfeeding support and experiences; weaning and complementary feeding; and type of birth delivery. Interviews lasted approximately 30 min each, were audio‐taped with participant permission, and were transcribed verbatim. Responses were recorded utilizing electronic case report forms in REDCap (version 8.1.1). De‐identified data were uploaded to a private password‐protected server. All participants provided written informed consent and received a $25 gift card honorarium as an expression of gratitude.

### 2.4 Ethics Statement

Ethical approval was obtained from the corresponding author's institutional research ethics board. A research agreement with the Department of Health and Social Services, Government of the Northwest Territories, and a Northwest Territories research license were obtained.

### 2.5 Data Analysis

Descriptive statistics, including frequencies and proportions, were generated. For categorical data, the Fisher's exact test was used. Quantitative analysis was performed using SAS statistical software (SAS Version 9.4, SAS Institute Inc. Cary, NC). Qualitative data were analyzed using an open coding method (Straus and Corbin 1998). After several rounds of reading and familiarization with the qualitative data, data were analyzed through reflexive thematic analysis, utilizing NVivo Pro version 12 (QSR International Pty Ltd., 2018) (Braun and Clarke 2006; Meyer and Ward 2014). Two analysts independently performed the initial coding. Any coding disagreements were resolved through discussion with another team member before the coding was finalized. The codes were collated into connective themes for interpretation. Qualitative research quality standards were maintained by following the Trustworthiness Criteria and Consolidated Criteria for Reporting Qualitative Research (COREQ) standards (Tong et al. 2007). A Triangulation Protocol was followed to integrate the quantitative and qualitative data sets to obtain a comprehensive understanding of the data.

## 3 Results

### 3.1 Demographics

Of the 145 participants (mean age 29.78 years; SD = ± 6.08), 12% were currently pregnant, 59% reported 2–4 past pregnancies, and 15% reported 5 or more past pregnancies. Approximately half (46%) became mothers for the first time before 20 years of age, and 35% became mothers between 20 and 29 years of age. The most common self‐identified ancestry was First Nations (52%), followed by Inuit (32%), and Métis (7%). Most participants (55%) had a high school diploma or post‐secondary education; 27% were employed full‐time, and 69% had the support of a spouse or partner (Table 1).

**TABLE 1:** Demographic characteristics of Indigenous mothers who had given birth in the last 3 years (*n* = 145).

Community	A	B	C	All
	*n* (%)	*n* (%)	*n* (%)	*n* (%)
Number of participants	22 (100)	64 (100)	5 (100)	145 (100)
Age (years)				
< 25	1 (4.55)	13 (20.31)	14 (23.73)	28 (19.31)
25–35	14 (63.64)	43 (67.19)	35 (59.32)	92 (63.45)
> 35	7 (31.82)	8 (12.5)	10 (16.95)	25 (17.24)
Mean age‐years (SD)^a^				29.78 (6.08)
Pregnant				
Yes	1 (4.55)	9 (14.06)	7 (11.86)	17 (11.72)
No	20 (90.91)	54 (84.32)	52 (88.14)	126 (86.9)
Number of pregnancies				
1	1 (4.55)	9 (14.06)	8 (13.56)	18 (12.41)
2–4	11 (50.00)	39 (60.94)	36 (61.02)	86 (59.31)
5–7	5 (22.73)	6 (9.38)	7 (11.86)	18 (12.41)
> 7	0	2 (3.13)	2 (3.39)	4 (2.76)
Missing	5 (22.73)	8 (12.5)	6 (10.17)	19 (13.1)
Age (years) at first pregnancy				
< 20	10 (45.45)	28 (43.75)	29 (49.15)	67 (46.21)
20–29	4 (18.18)	29 (45.31)	18 (30.51)	51 (35.17)
30–39	1 (4.55)	0	7 (11.86)	8 (5.52)
Missing	7 (31.82)	7 (10.94)	5 (8.47)	19 (13.1)
Ethnicity^b^				
First Nations	22 (100.00)	16 (25)	37 (62.71)	75 (51.72)
Inuit	0	40 (62.5)	7 (11.86)	47 (32.41)
Métis	0	0	10 (16.95)	10 (6.9)
Inuit and First Nations	0	5 (7.81)	3 (5.08)	8 (5.52)
Education				
Post‐secondary education^c^	3 (13.64)	25 (39.06)	25 (42.37)	53 (36.55)
High school diploma or equivalent	2 (9.09)	11 (17.19)	13 (22.03)	26 (17.93)
Less than or some high school	17 (77.27)	28 (43.75)	20 (33.9)	65 (44.83)
Employment status^b^				
Full time	4 (18.18)	16 (25)	19 (32.2)	39 (26.9)
Maternity leave	2 (9.09)	8 (12.5)	8 (13.56)	18 (12.41)
Part‐time	4 (18.18)	8 (12.5)	2 (3.39)	14 (9.66)
Student	0	5 (7.81)	3 (5.08)	8 (5.52)
Not working^d^	12 (54.55)	26 (40.6)	27 (45.76)	65 (44.83)
Currently have a partner or spouse				
No	4 (18.18)	18 (28.13)	20 (33.9)	42 (28.97)
Yes	17 (77.27)	45 (70.31)	38 (64.41)	100 (68.97)

^a^ SD (standard deviation).

^b^ Missing data or data levels (such as “unsure” or “don't know”) with less than five observations were omitted.

^c^ Completed any post‐secondary, including vocational or cultural training, college diploma, or university degree.

^d^ Not working includes the following response options: not working and looking; not working and not looking; and unable to work.

### 3.2 Quantitative Results

A summary of infant feeding intentions and practices is found in Table 2. During pregnancy, 73% of participants had intended to breastfeed exclusively, 9% had intended to formula feed, and 17% had planned to do a combination of breastfeeding and formula feeding. After birth, 87% of participants initiated breastfeeding, with babies placed to the breast within 30 min of delivery for 48% of mothers, within 2 h of delivery for 35% of participants, and ≥ 24 h after delivery for 8% of participants. Regarding feeding, 44% of participants breastfed exclusively (either feeding at the breast or expressed) for < 3 months, 29% for 3–6 months, 15% for 6–11 months, and 5% for 12 months or more, after which complementary feeding including baby formula, water, infant cereal, juice, and tea was introduced. Overall, 82% of participants received support at the hospital or health centre with breastfeeding initiation, and 48% reported no challenges with breastfeeding.

**TABLE 2:** Infant feeding intentions and practices of Indigenous mothers who had given birth in the last three years (*n* = 145).

Community	A	B	C	All
	*n* (%)	*n* (%)	*n* (%)	*n* (%)
Future infant feeding intentions during pregnancy^a^				
Breastfeeding only (including pumped)	10 (45.45)	50 (78.13)	46 (77.97)	106 (73.1)
Formula feeding only	5 (22.73)	4 (6.25)	4 (6.78)	13 (8.97)
Combination of both	7 (31.82)	8 (12.5)	9 (15.25)	24 (16.55)
Did you initiate breastfeeding?				
Yes	15 (68.18)	57 (89.06)	54 (91.53)	126 (86.9)
No	7 (31.82)	7 (10.94)	5 (8.47)	19 (13.1)
Approximate time after birth the baby was first put to the breast^a^, ^b^				
Within 30 min	6 (40)	32 (56.14)	22 (40.74)	60 (47.62)
Within 2 h	6 (40)	16 (28.07)	22 (40.74)	44 (34.92)
Within 12 h	1 (6.67)	2 (3.51)	4 (7.41)	7 (5.56)
Within 24 h	0	0	1 (1.85)	1 (0.79)
After 24 h or more	2 (13.33)	3 (5.26)	4 (7.41)	9 (7.14)
Exclusive breastfeeding^a^, ^b^				
< 3 months	4 (26.67)	27 (47.37)	25 (46.3)	56 (44.44)
3**–**6 months	7 (46.67)	16 (28.07)	13 (24.07)	36 (28.57)
6**–**11 months	3 (20)	8 (14.04)	8 (14.81)	19 (15.08)
≥ 12 months	0	4 (7.02)	2 (3.7)	6 (4.76)
Still breastfeeding	1 (6.67)	1 (1.75)	3 (5.56)	5 (3.97)
Support received to initiate breastfeeding at the hospital or health center^a^, ^b^				
Yes	11 (73.33)	52 (91.23)	40 (74.07)	103 (81.75)
No	4 (26.67)	3 (5.26)	11 (20.37)	18 (14.29)
Any challenges with breastfeeding ^b^				
Yes	8 (53.33)	31 (54.39)	26 (48.15)	65 (51.59)
No	7 (46.67)	26 (45.61)	28 (51.85)	61 (48.41)
Approximate age of the baby when you weaned or stopped breastfeeding^b^				
< 3 months	1 (6.67)	10 (17.54)	12 (22.22)	23 (18.25)
> 3 < 6 months	2 (13.33)	3 (5.26)	6 (11.11)	11 (8.73)
> 6 < 12 months	0	6 (10.53)	6 (11.11)	12 (9.52)
> 12 months	1 (6.67)	12 (21.05)	6 (11.11)	19 (15.08)
Still breastfeeding	9 (60)	23 (40.35)	21 (38.89)	53 (42.06)
Missing	2 (13.33)	3 (5.26)	3 (5.56)	8 (6.35)
Approximate age when the infant first had solid food^c^				
< 3 months	0	2 (3.13)	2 (3.39)	4 (2.76)
> 3 < 6 months	5 (22.73)	11 (17.19)	12 (20.34)	28 (19.31)
> 6 < 12 months	11 (50)	33 (51.56)	28 (47.46)	72 (49.66)
> 12 months	1 (4.55)	4 (6.25)	2 (3.39)	7 (4.83)
Still breastfeeding	1 (4.55)	1 (1.56)	2 (3.39)	4 (2.76)
Not yet given	3 (13.64)	10 (15.63)	9 (15.25)	22 (15.17)
Missing	1 (4.55)	2 (3.13)	3 (5.08)	6 (4.14)
Approximate age when the infant was first given traditional food or medicine				
< 3 months	2 (9.09)	0	2 (3.39)	4 (2.76)
3–6 months	7 (31.82)	24 (37.5)	14 (23.73)	45 (31.03)
6–12 months	6 (27.27)	19 (29.69)	21 (35.59)	46 (31.72)
12–24 months	0	2 (3.13)	1 (1.69)	3 (2.07)
Missing	7 (31.82)	19 (29.69)	21 (35.59)	47 (32.41)
Direct skin‐to‐skin contact the first time the baby was held^a^				
Yes	18 (81.82)	51 (79.69)	41 (69.49)	110 (75.86)
No	4 (18.18)	13 (20.31)	17 (28.81)	34 (23.45)
The place where the baby was during the first hour after birth^a^				
Skin‐to‐skin	13 (59.09)	41 (64.06)	31 (52.54)	85 (58.62)
In bed with the mother	2 (9.09)	8 (12.5)	5 (8.47)	15 (10.34)
In the room with the mother	2 (9.09)	7 (10.94)	8 (13.56)	17 (11.72)
Not in the same room	5 (22.73)	5 (7.81)	11 (18.64)	21 (14.48)
Unsure/Don’t know	0	2 (3.13)	3 (5.08)	5 (3.45)

^a^ Missing data or data levels (such as ‘unsure’ or ‘don’t know’) with less than five observations were omitted.

^b^ Responses for mothers who did breastfeed or attempted to (*n* = 126).

^c^ Solid foods: defined as pureed, mashed, strained, or soft foods that are not liquid.

As well, 15% of participants breastfed for ≥ 12 months, with solid foods (pureed, mashed, strained, or soft foods that are not liquid) being introduced by 19% of participants between 3 and 6 months and 50% of participants between 6 and 12 months. Most participants (66%) provided traditional medicine or food before 12 months. Regarding contact, during the first hour after birth, 59% of participants had skin‐to‐skin contact with the baby, 10% had the baby sharing the bed, 12% had the baby in the room, and 15% were not in the same room as the baby. Overall, 76% of the participants had skin‐to‐skin contact when holding the baby for the first time (Table 2). Rates of skin‐to‐skin contact and rooming‐in were significantly higher (*p* < 0.0001) among participants who had experienced vaginal births compared to emergency and scheduled Caesarean sections (Table 3).

**TABLE 3:** Frequency of skin‐to‐skin contact and rooming‐in based on the type of childbirth for Indigenous mothers who had given birth in the last three years (*n* = 145).

	Type of childbirth			
	Vaginal birth	Emergency caesarean–section	Scheduled caesarean–section	Total
	*n* (%)	*n* (%)	*n* (%)	*n* (%)
Direct skin‐to‐skin contact the first time the baby was held (*p*‐value < 0.0001)^a^, ^b^				
Yes	99 (87.61)	6 (31.58)	5 (38.46)	110 (75.86)
No	14 (12.39)	12 (63.16)	8 (61.54)	34 (23.45)
The place where the baby was during the first hour after birth (*p*‐value < 0.0001)^a^, ^b^				
Skin‐to‐skin	78 (69.03)	4 (21.05)	3 (23.08)	85 (58.62)
In bed with the mother	15 (13.27)	0	0	15 (10.34)
In the room with the mother	8 (7.08)	5 (26.32)	4 (30.77)	17 (11.72)
Not in the same room	8 (7.08)	8 (42.11)	5 (38.46)	21 (14.48)

^a^ Fisher's exact test.

^b^ Missing data or data levels (such as ‘unsure’ or ‘don’t know’) with less than five observations were omitted.

### 3.3 Qualitative Results

Three themes were identified: barriers to breastfeeding, facilitators of breastfeeding, and formula feeding.

#### 3.3.1 Theme 1: Barriers to Breastfeeding

This theme denotes the barriers to breastfeeding. Some participants described poor latching on and related physical challenges in newborns (e.g., cleft palate) as barriers. When latching on was not established, some participants reported expressing breast milk.

I really wanted to breast feed them. It was a huge struggle. I heard that breastfeeding was hard but didn't anticipate how hard and emotional it would be

Baby wouldn't latch

I wanted to breast feed but I couldn't because he couldn't latch on due to his cleft palate

Pumped breastmilk only. She could not latch

During the first few days of the post‐partum period, some participants reported babies were hungry, dissatisfied with human milk, and/or not receiving enough nutrition.

When my baby was born he was hungry and I had to give him a bottle. Cause my milk wasn't in yet

Low human milk production was also described as a barrier to breastfeeding. Remedies included pharmacotherapy, expressing human milk, maternal dietary supplementation, and increased water intake.

I took supplements and was drinking upwards of 10L of water a day. I made special cookies, I made special smoothies. I did everything I could to make milk. I pumped and I pumped and I pumped and I got like 2oz. of milk

Physical discomfort was also a barrier to breastfeeding for some participants.

Sore nipples, swelling, agony

#### 3.3.2 Theme 2: Facilitators of Breastfeeding

This theme denotes facilitators of breastfeeding. Many participants reported that being supported by family, friends, healthcare professionals, and/or traditional healers facilitated the initiation and continuation of breastfeeding.

My family helped a lot

My friend was right there helping me too

from a public health nurse, who was excellent. And my family. And a healer

For instance, participants described healthcare professionals providing advice on breastfeeding technique, encouragement, and respite as facilitators of breastfeeding. Participants also reported that community‐based organizations provided needed advice and support through help lines and informational brochures.

They showed me how I should be breastfeeding

nurses had other techniques that I could try. That's why I really felt supported by the nurses at the hospital

The nurse would not let me give up

They showed me on which part and how to hold him while breastfeeding. Showed me how to get him to latch on

They just re‐showed me how to breastfeed. Different techniques

They took the baby so I could nap

Moms Boobs and Babies has a help line, or a support line. They're actually really good….had lots of good suggestions and support

Public health nurses who made home visits were described as a source of motivation, support, and advice for breastfeeding, and provided participants additional supplies such as nipple shields, barrier creams, and prescription medication to improve lactation.

I was given a prescription to help get my milk going. The nurse suggested that I pump in between. Breast, formula, pump…The nurse had to come 3–4 times per week to check on his weight

I think they're called nipple shields, just make it so the little babies can latch on, and when I put that on he instantly was able to latch and start drinking and like, the tears just flowed

The public health nurse came to my house a few days after. She gave me some cream for cracked nipples

Additional facilitators of breastfeeding reported by participants included previous breastfeeding experience, the health benefits for both mother and baby, and the convenience of feeding from the breast.

she latched right away. The doctor was like “you're pro” and I was like, “its her too.” First child it was harder

It feels comfortable and lose weight very fast. Healthy for baby and me too. Saving the time.

I've always wanted to breastfeed, just because of the benefits

Giving the bottle wastes too much time.

#### 3.3.3 Theme 3: Formula Feeding

This theme denotes factors that resulted in initiating formula feeding. Emotional challenges after childbirth, low human milk production, increased infant feeding demands, limited assistance with breastfeeding, and infants being away from participants when in the care of fathers were reasons given by participants for the introduction of formula feeding.

like I didn't feel like myself for like a really long time after he was born, and so I had really low milk supply so I switched to formula

she was emptying me like crazy but she still was not satisfied. So a week later I ended up introducing formula to her

I was planning on just breastfeeding but I felt like I was not able to produce enough milk. After the first week we slowly started to introduce formula. About a month after he was born he was on just straight formula

Right now she is bottle fed when she goes to see her Dad. Maybe twice a week

I didn't receive any help, I just ended up giving him formula

One participant reported that her baby had been introduced to formula feeding by a nurse when she was asleep, resulting in subsequent difficulties with breastfeeding.

I wanted to breastfeed but the nurse gave the baby formula when I was sleeping. The baby got used to the formula and now it's hard to breastfeed

## 4 Discussion

This project explored the infant feeding intentions and experiences of Indigenous mothers in NWT. While pregnant, most participants had intended to breastfeed exclusively. Many participants also initiated breastfeeding; however, rates of exclusively breastfeeding steadily declined by six months post‐partum. Breastfeeding technique and low human milk production were barriers to breastfeeding. Receiving support and informational resources from family and healthcare professionals were facilitators of breastfeeding.

The rate of breastfeeding initiation found in this project was slightly lower than the national estimate of 90%, but similar to the estimate for the Canadian territories (88%) (Public Health Agency of Canada 2022). However, the steady decline in exclusive breastfeeding within the first six months reported in this project is of concern. Factors that influenced the decline included emotional challenges after childbirth, low human milk production, increased infant feeding demands, limited assistance with breastfeeding, and infants being away from mothers. Firstly, breastfeeding is an important aspect of food security for remote communities in Northern Canada where market food is expensive and traditional country food is becoming less accessible (Willows 2013). Secondly, the premature discontinuation of breastfeeding can lead to emotional distress for mothers (Hegney et al. 2008) and increase the risk of illness and disease for mothers and infants (McIsaac et al. 2015). It should be noted that breastfeeding is affected by the length of a mother's maternity leave, with a delayed return to work increasing the duration of breastfeeding (Ogbuanu et al. 2011). Participants returning to work may have contributed to the premature discontinuation of exclusive breastfeeding. Regardless, most participants introduced complementary feeding at the recommended age of six months (Nickel et al. 2014), with many also introducing traditional foods and medicines before 12 months of age. The inclusion of traditional foods during weaning should be encouraged, as infants establish taste and texture preferences during these first months (Borowitz 2021).

Low human milk production during the first few days after childbirth was reported by many participants as a reason for discontinuing breastfeeding and introducing formula feeding. After childbirth, colostrum is released and then, from the second day after delivery and for up to two weeks, transitional milk is released before being replaced with mature human milk (Thakkar et al. 2019). Frequent feeding by infants during the first few days after birth helps establish the transition to human milk. As such, contact with newborns shortly after delivery should be encouraged for all mothers irrespective of the route of delivery. Some mothers may not be aware of this process, resulting in the premature discontinuation of breastfeeding, and providing informational resources regarding the phases of human milk may help lower rates of premature breastfeeding discontinuation.

Access to care limitations with medical and dental specialists can play a critical role in the feeding practices of infants with craniofacial abnormalities such as cleft lip and palate, which have been shown to have the highest incidence in Indigenous populations (Lowry et al. 2009; Vrouwe et al. 2013; Wolfswinkel et al. 2022). The American Cleft Palate‐Craniofacial Association's Parameters for the Evaluation and Treatment of Patients with Cleft Lip/Palate or Other Craniofacial Anomalies recommends access to an interdisciplinary team whose first responsibility is to provide assistance with feeding, including weekly nutritional monitoring and assessment of weight gain (American Cleft Palate‐Craniofacial Association 2017). Surgical repair should ideally occur within the first twelve months, which coincides with and therefore may affect the period of highest frequency of breastfeeding.

Supporting breastfeeding mothers is a societal responsibility where all aspects of the society, including the community, healthcare professionals, employers, and family members, are important for creating an environment that enables and supports breastfeeding (Rollins et al. 2016). Women in this project received support at hospital and home visits by nurses, which helped with breastfeeding in multiple ways, including motivation, support, information, and supplies/medications. This is in line with the Baby‐Friendly Initiative (BFI) and reinforces the current practices that are helping Indigenous community members. The Department of Health and Social Services (DHSS) has supported the implementation of the BFI in facilities across NWT, with the Inuvik Regional Hospital becoming a Baby‐Friendly facility in December 2018. BFI was developed by the World Health Organization (WHO) and United Nations Children's Fund (UNICEF) and aims to ensure that all mothers and infants receive the highest quality of family‐centered care, which includes information about breastfeeding and infant feeding support (*World Health Organization and* *UNICEF* 2009). However, breastfeeding is most successful as a family‐centered activity that distributes the responsibility of breastfeeding (Ke et al. 2018), with partners providing emotional support and participating in decision making (de Montigny et al. 2018; Ke et al. 2018). Medical travel from remote Northern communities over long distances for childbirth (Silver et al. 2022) may prevent Indigenous mothers from experiencing such continuous cultural support from community and family members (Smylie 2014). Further supporting Indigenous mothers in breastfeeding for greater durations utilizing a family‐centered and culturally sensitive approach, which includes support and information for traditional infant feeding practices, is warranted. The utilization of web‐based interventions in providing such support (Lau et al. 2016) also warrants further investigation, with social media (Brown 2016) and smartphone apps (Coughlin 2016) having been successfully used to promote breastfeeding in other settings.

A mother returning to work often presents a barrier to extended exclusive breastfeeding; maternity leave ensures a source of income and is crucial for promoting exclusive breastfeeding in the first six months of a child's life (Rimes et al. 2019). For instance, in Nordic and Eastern European countries, longer maternity leave periods (49 weeks at full pay) for working mothers facilitated extended breastfeeding (Strang and Broeks 2017). Providing longer paid maternity leave and facilitating supportive work environments with breastfeeding facilities are encouraged.

The Aurora College in NWT has integrated the BFI infant feeding and family‐centered maternity care learning objectives into its nursing program, equipping graduates with the knowledge of infant feeding support. Continuing to train healthcare professionals on culturally sensitive infant feeding knowledge and skills, as well as timely skin‐to‐skin contact, is recommended. Utilizing a participatory approach with Indigenous communities to implement culturally sensitive strategies to address infant feeding practices and skills is recommended. Such support can also take the form of online resources, which often reach a wider audience and are convenient. This paper provided valuable insight into the infant feeding practices and experiences of Indigenous mothers in NWT from communities with varying levels of healthcare infrastructure. Importantly, data were collected utilizing a culturally sensitive questionnaire developed in consultation with the CAB, which helped ensure the appropriateness and relevance of the questions within the local context. We used a triangulation approach to compare and contrast patterns from qualitative and quantitative data, enhancing the depth and credibility of the findings. This integration provided both empirical strength and contextual insight, leading to a more holistic understanding of the research problem. Several limitations should be acknowledged. First, interviews were based on the self‐report of mothers, many of whom were motivated to breastfeed. This may have introduced the potential for social desirability bias, as participants may have reported behaviors deemed favorable or expected rather than actual practice. Second, the convenience sampling methods utilized in this project limit the generalizability of results. Additionally, recall bias may have influenced the accuracy of responses, particularly for mothers recalling events during the postpartum period. The results of Fisher's Exact Test regarding skin‐to‐skin contact and rooming‐in were presented at the bivariate level. This approach limited the ability to control for potential confounding or intervening variables. Consequently, the observed associations should be interpreted with caution, as they may be influenced by underlying factors that were not included in the analysis, such as maternal age.

### 4.1 Conclusion

Many Indigenous mothers in NWT successfully initiate breastfeeding, yet many face challenges sustaining breastfeeding beyond three months. To enhance breastfeeding support within Indigenous communities, it is essential to adopt a community‐led approach that leverages local expertise and traditional knowledge. Emphasizing an intergenerational perspective that involves the wisdom of community Elders may greatly strengthen efforts. This paper is a valuable resource for designing and implementing policies that promote early infant nutrition and breastfeeding within Indigenous communities, both in NWT and elsewhere in Canada.

## Author Contributions

**Rachel Harris:** formal analysis (equal), validation (equal), writing – original draft (lead), writing – review and editing (equal). **Fariba Kolahdooz:** conceptualization (equal), supervision (equal), validation (equal), writing – review and editing (equal). **Radha Sharma:** formal analysis (equal), validation (equal), writing – review and editing (equal). **Moutasem Zakkar:** formal analysis (equal), validation (equal), writing – review and editing (equal). **Adrian Wagg:** validation (equal), writing – review and editing (equal). **André Corriveau:** visualization (equal), writing – review and editing (equal). **Marie Tarrant:** validation (equal), writing – review and editing (equal). **Stephanie Irlbacher‐Fox:** validation (equal), writing – review and editing (equal). **Tyler Verhaeghe:** validation (equal), writing – review and editing (equal). **Sangita Sharma:** conceptualization (equal), funding acquisition (lead), supervision (equal), validation (equal), writing – review and editing (equal).

## Ethics Statement

The Research Ethics Board at the University of Alberta issued the research ethics certificate. Following the Scientists Act of Northwest Territories, researchers obtained a research license from the Aurora Research Institute.

## Consent

Written informed consent was obtained from all participants.

## Conflicts of Interest

The authors declare no conflicts of interest.

## Data Availability

All data supporting the findings of this study are available within the paper.
